# An overview of obesity‐related complications: The epidemiological evidence linking body weight and other markers of obesity to adverse health outcomes

**DOI:** 10.1111/dom.16263

**Published:** 2025-03-11

**Authors:** Matthias Blüher

**Affiliations:** ^1^ Helmholtz Institute for Metabolic, Obesity and Vascular Research (HI‐MAG) of the Helmholtz Zentrum München University of Leipzig and University Hospital Leipzig Leipzig Germany; ^2^ Medical Department III—Endocrinology, Nephrology, Rheumatology University of Leipzig Medical Center Leipzig Germany

**Keywords:** adipose tissue, cardiometabolic diseases, complications, obesity

## Abstract

Obesity is a highly prevalent chronic multisystem disease associated with shortened life expectancy due to a number of adverse health outcomes. Epidemiological data link body weight and parameters of central fat distribution to an increasing risk for type 2 diabetes, hypertension, fatty liver diseases, cardiovascular diseases including myocardial infarction, heart failure, atrial fibrillation, stroke, obstructive sleep apnoea, osteoarthritis, mental disorders and some types of cancer. However, the individual risk to develop cardiometabolic and other obesity‐related diseases cannot entirely be explained by increased fat mass. Rather than excess fat accumulation, dysfunction of adipose tissue may represent the mechanistic link between obesity and adverse health outcomes. There are people living with obesity who seem to be protected against the premature development of cardiometabolic diseases. On the other hand, people with normal weight may develop typical obesity diseases upon dysfunction of adipose tissue and predominantly visceral fat distribution. The mechanisms linking impaired function of adipose tissue in people with obesity include adipocyte hypertrophy, altered cellular composition, limited expandability of safe subcutaneous fat stores, ectopic fat deposition in visceral depots, the liver and other organs, hypoxia, a variety of stresses, inflammatory processes, and the release of pro‐inflammatory, diabetogenic and atherogenic signals. Genetic and environmental factors might contribute either alone or via interaction with intrinsic biological factors to variation in adipose tissue function. There are still many open questions regarding the mechanisms of how increased body weight causes obesity‐related disorders and whether these pathologies could be reversed. Evidence‐based weight loss interventions using behaviour change, pharmacological or surgical approaches have clarified the beneficial effects of realistic and sustained weight loss on obesity‐related complications as hard outcomes. This review focusses on recent advances in understanding epidemiological trends and mechanisms of obesity‐related diseases.

**Plain Language Summary:**

Obesity is a chronic complex and progressive disease characterized by excessive fat deposition that may impair health and quality of life. Worldwide, the number of adults living with obesity has more than doubled since 1990. Obesity may lead to reduced life expectancy, because it increases the risk for type 2 diabetes, cardiovascular diseases (e.g., myocardial infarction, high blood pressure, stroke), fatty liver diseases, musculoskeletal diseases, chronic respiratory diseases, depression and certain types of cancer. However, not every person with obesity develops these diseases. For better prevention and treatment, it is important to understand the mechanisms linking high fat mass to obesity related diseases. It has become clear that fat mass alone cannot explain the higher risk of obesity complications. People with obesity can have either high or low risk of developing complications. Compared to people with a low risk for obesity complications those with a high risk to develop obesity related diseases are characterized by higher central fat deposition in the abdominal region, on average bigger fat cells, higher number of immune cells in adipose tissue and altered signals released from adipose tissue that may directly affect the brain, liver, vasculature and other organs. Both inherited and environment factors may cause these abnormalities of adipose tissue function. However, weight loss through behaviour changes (e.g., lower calorie intake, higher physical activity), medications or obesity surgery can improve health, quality of life and reduce the risk for obesity related diseases.

## 1 INTRODUCTION

Obesity is a chronic, relapsing, non‐communicable multisystem disease characterised by an abnormal and/or excessive accumulation of body fat that presents a risk to health.^1^, ^2^

**FIGURE 1:**
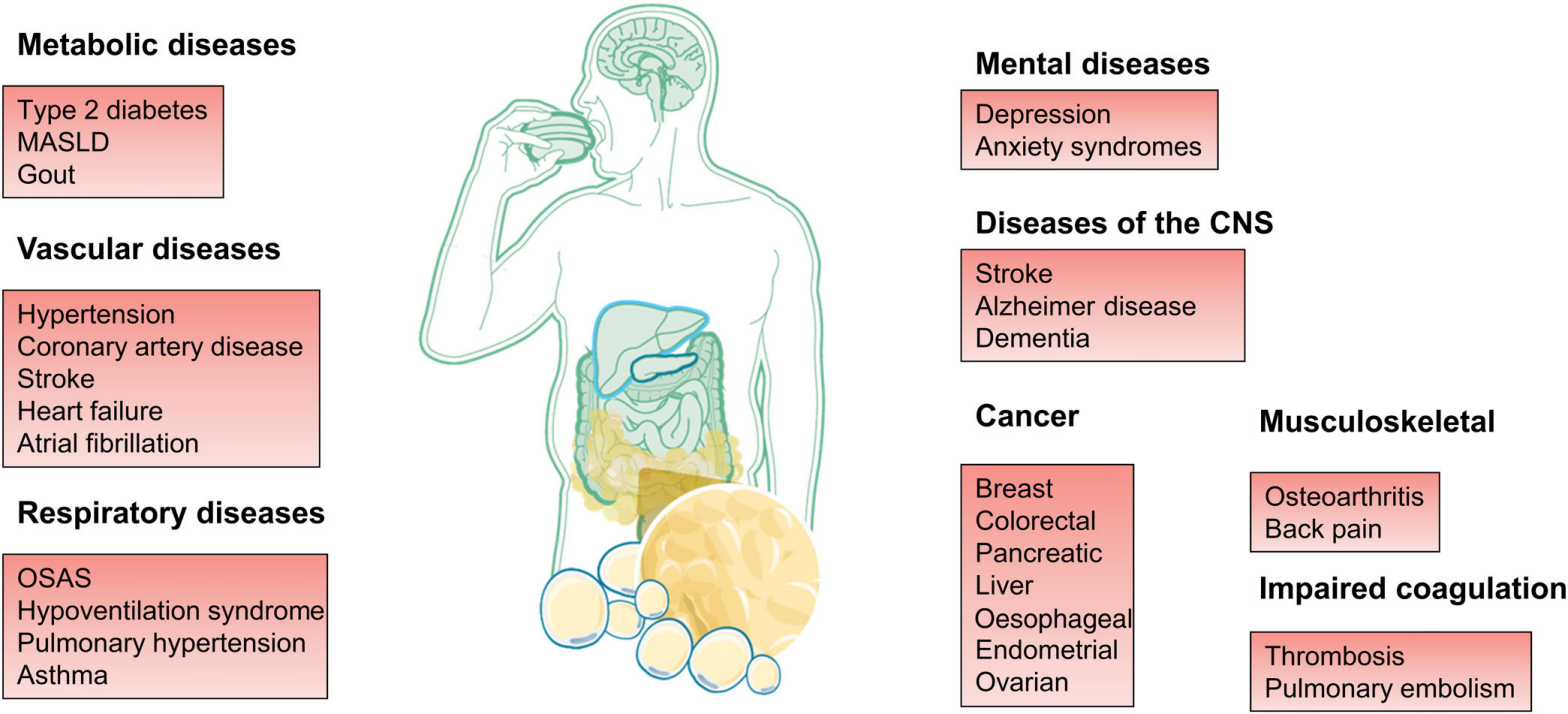
Obesity as a multisystem disease. Selected complications that are most strongly related to obesity. MAFLD, metabolic dysfunction‐associated fatty liver disease; OSAS, obstructive sleep apnea syndrome.

Obesity impairs quality of life and contributes to a reduced life expectancy mainly because of several related complications including type 2 diabetes, hypertension, fatty liver diseases, cardiovascular diseases including myocardial infarction, heart failure, atrial fibrillation, stroke, obstructive sleep apnoea, osteoarthritis, mental disorders and some types of cancer (Figure 1).^3^, ^4^, ^5^ The fundamental cause of obesity is a long‐term energy imbalance between too many calories consumed and too few calories expended.^3^ Obesity causes are complex and include environmental, behavioural, biological factors and their interplay.^3^ In addition to an obesogenic environment, biological mechanisms may cause obesity or increase the susceptibility to the development of obesity under modern living conditions.^3^ In this context, the brain plays a central role in the pathogenesis of obesity, because it regulates energy metabolism, food intake and energy expenditure.^3^ Altered neurocircuits, in different brain areas such as the hypothalamus and changes of the cross‐talk between the brain and peripheral organs (e.g., adipose tissue, liver, gut, pancreas) underlie the chronic positive energy balance that leads to obesity.^3^

According to the World Health Organization, in the year 2022, 16% of people (890 million) in the world were living with obesity.^1^ Five percent of worldwide mortality were attributable to obesity and its related diseases.^6^ The prevalence of obesity in adults has more than doubled since 1990, and adolescent obesity has quadrupled.^1^ Over 160 million children were living with obesity in 2022.^1^ The prevalence of obesity is generally higher in women than men, increasing with age after the age of 14 years, and is higher in countries with a high sociodemographic index.^3^, ^5^ Despite the increasing obesity prevalence in all countries, there are regional differences that may help us to better understand the main drivers of the disease and to adjust treatment recommendations to regionally distinct conditions.^3^

Obesity constitutes an enormous burden to societies because of its associated public health and economic burden.^6^, ^7^ In addition to excess health care expenditure, obesity and associated health impairments are associated with high indirect costs through lost productivity and slower economic growth, lost working days, lower productivity at work, mortality and permanent disability.^6^

Although now widely recognised as a disease, it is clear that more needs to be done to provide structured assessments and interventions tailored to the individual person living with obesity.

### 1.1 Diagnosis of obesity

In the clinical setting, the diagnosis of obesity is still based on body mass index (BMI) cut‐off values alone.^2^ Indications for obesity treatment are mostly based on anthropometric measurements, rather than on a more complete clinical evaluation of the individual obesity‐related risk.^2^ Recently, a Lancet Commission on clinical obesity proposed criteria as new diagnostic criteria for obesity as a disease.^8^ The commission suggests to pragmatically distinguish clinical obesity from preclinical obesity on the basis of the presence of objective clinical manifestations of altered organ function or impairment of an individual's ability to conduct daily activities.^8^ In this framework, preclinical obesity is considered a state of excess adiposity with preserved function of other tissues and organs and a varying, but generally increased, risk of developing clinical obesity and several other non‐communicable diseases.^8^ In contrast, ‘clinical obesity’ can lead to severe end‐organ damage, causing life‐altering and potentially life‐threatening complications.^8^ Background for the Lancet Commission consensus was the narrative that BMI‐based measures of obesity can both underestimate and overestimate adiposity and inadequately reflect the health status at the individual level.^8^

However, current obesity management guidelines still define obesity by a BMI of >30 kg/m^2^ and for Asian people by a BMI >25 kg/m^2^.^1^, ^9^

In contrast, the Lancet Commission recommends that BMI should be used only as a surrogate measure of health risk at a population level, for epidemiological studies, or for screening purposes, rather than as an individual measure of health.^8^ Excess adipose tissue (AT) accumulation should be confirmed by either direct measurement of body fat or at least one anthropometric criterion including waist circumference, waist‐to‐hip ratio, or waist‐to‐height ratio in addition to BMI. Only in people with very high BMI >40 kg/m^2^, excess adiposity can pragmatically be assumed, and no further confirmation is required.^8^

**FIGURE 2:**
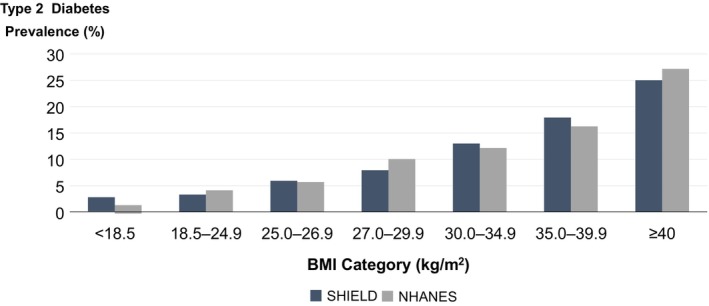
Association between type 2 diabetes and body mass index (BMI) categories. Data from the US Study to Help Improve Early evaluation and management of risk factors Leading to Diabetes (SHIELD) and National Health and Human Nutrition Examination Surveys (NHANES) revealed that with increasing BMI, the frequency of type 2 diabetes increases.^9^, ^24^

In general, the risk for obesity‐related disorders increases with increasing BMI. The association between BMI and cardiometabolic diseases is particularly pronounced for the risk to develop type 2 diabetes (Figure 2)^10^, ^11^ and hypertension.^12^, ^13^ Compared to a person with a BMI <25 kg/m^2^, the obesity‐related risk to develop type 2 diabetes increases from <5% to >25% (Figure 2). However, the relationships between BMI and the risk for distinct obesity complications are not always linear and the flipping point at which the obesity‐related disease risk increases is variable.^4^, ^13^ Indeed, body weight and BMI do not always reflect increased body fat mass, AT dysfunction and fat distribution, which are better predictors of cardiometabolic disease risk upon fat accumulation.^13^, ^14^, ^15^, ^16^ Waist circumference and/or waist‐to‐hip ratio as surrogate parameters for fat distribution are better predictors of mortality and cardiometabolic morbidity in people with obesity.^16^, ^17^ At any given BMI, the variation in comorbidities and health risk factors is remarkably high.^17^ Noteworthy, Asian populations and some other ethnic groups develop complications typical for obesity at a lower BMI compared to people with European or African ethnic background.^18^, ^19^ The ethnic differences in the development of obesity complications have been mainly attributed to differences between populations in body composition and fat distribution.^19^ In Asians, increasing BMI is associated with larger increases in body fat mass and abdominal fat distribution, but these factors alone cannot explain ethnic disparities in the onset, severity and progress of obesity complications. Mechanisms including altered adipokine secretion, impaired insulin secretion upon metabolic stimuli and earlier onset of adipocyte maturation arrest as well as ‘premature insulin resistance’ during weight gain could be factors increasing the cardiometabolic risk of Asian populations at a lower BMI.^20^

To improve the current shortcomings in therapeutic indications, targets and the choice of the type and intensity of treatment, the European Association for the Study of Obesity (EASO) recently proposed a new framework for the diagnosis, staging and management of obesity in adults to better align with the concept of obesity as an adiposity‐based chronic disease.^2^

In the proposed framework, it is highlighted that abdominal fat accumulation is a stronger determinant for developing cardiometabolic complications than BMI—even in people with normal weight or overweight.^21^ In line with the recent proposal from the Lancet Commission on clinical obesity,^8^ the EASO statement further introduces the waist‐to‐height ratio instead of waist circumference as superior indicator of cardiometabolic disease risk.^22^

In addition to the assessment of body weight, BMI and parameters of fat distribution, the clinical diagnostic of obesity should include a systematic evaluation of medical, functional and psychological impairments, an approach that EASO adopted from other guidelines and the Edmonton Obesity Staging System.^23^, ^24^, ^25^ Within data from the National Health and Human Nutrition Examination Surveys (NHANES) 1988–1994, the Edmonton obesity staging system was a better predictor of mortality compared to obesity classes based on BMI categories even after adjustment for other measures of adiposity.^26^

Importantly, psychological factors, depression, anxiety, reduced quality of life and other psychosocial factors have a large impact on obesity itself and may modulate the individual risk to develop obesity complications.^3^ Therefore, future obesity treatment strategies need to address patient‐centric outcomes, quality of life and psychological outcomes in addition to the physiological changes upon treatment.

In the past years, treatment options for the management of obesity improved significantly.^27^, ^28^ Despite novel treatment options, particularly in pharmacological interventions, evidence‐based obesity management therapies including behaviour change‐based, pharmacological or surgical therapies of obesity are under‐valued by clinicians and many health care systems.

### 1.2 Are obesity complications reversible?

The fundamental cause of obesity is an energy imbalance between calories consumed and calories expended.^2^ However, the aetiology of obesity is more complex and involves biological, genetic, environmental and societal factors.^2^ The complexity of the root causes in the pathogenesis of obesity may explain the challenges in the prevention and treatment of the disease. Despite these challenges and the notion that obesity is a chronic progressive disease, it can be treated by weight loss interventions.^27^ Behavioural, pharmacological and surgical interventions represent the main strategies for the management of obesity.

The definition of obesity as a chronic disease rather than a risk factor for related diseases is justified by its distinct pathophysiology that leads to excess and ectopic fat accumulation.^27^ Moreover, the body defends its body weight by homeostatic mechanisms that hinder weight loss and promote further weight gain.^29^ Altered biological mechanisms including changes in central neurocircuits and body–brain cross‐talk may explain why short‐term interventions aiming at behaviour changes are frequently not successful to provide a sustained long‐term weight loss.^27^, ^29^

**FIGURE 3:**
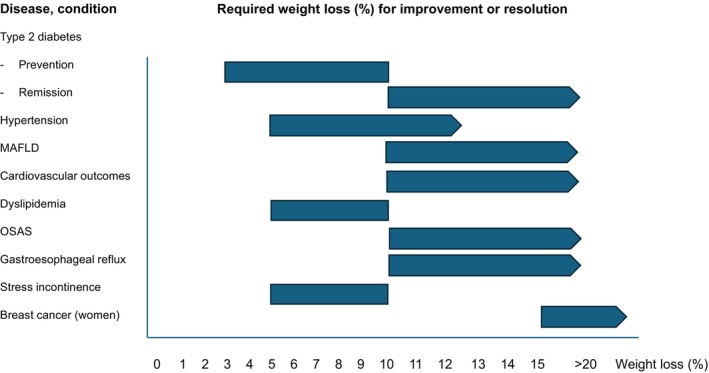
Effects of weight loss (%) on risk factors, and obesity‐related comorbidities and complications. The figure has been adopted from 78. MAFLD, metabolic dysfunction‐associated fatty liver disease; OSAS, obstructive sleep apnea syndrome.

Therefore, obesity treatment needs to be escalated by adding pharmacological and/or surgical interventions. With increasingly effective obesity therapies, obesity can be brought into remission in individual patients, but not yet at a large scale. Bariatric surgery represents the most effective treatment strategy reducing body weight and improving obesity‐related diseases. There is a large body of evidence that even with moderate weight loss, a number of obesity complications can be improved, prevented or a disease remission can be achieved (Figure 3).^30^ In the Swedish Obese Subjects Study, a sustained average weight loss of >25% led to increased life expectancy of approximately 3 years and decreased mortality from cardiovascular disease or cancer by 30% and 23%, respectively.^31^ These benefits of bariatric surgery‐induced weight loss were most likely attributable to long‐term improvements in blood pressure, glucose and lipid metabolism.^32^

The multiple benefits of weight loss further underline the view that obesity is a multisystem disease that affects the function of multiple biological processes, tissues and organs simultaneously.^4^ Weight loss in people with obesity has been shown to prevent, reduce or resolve certain comorbidities (Figure 3). In prospective clinical trials, behaviour‐based and pharmacotherapy treatments have been demonstrated to reduce the risk to develop type 2 diabetes in individuals with prediabetes.^33^, ^34^ Interestingly, the required weight loss to prevent or delay type 2 diabetes in more than half of the study participants of the Diabetes Preventions Study and the Finnish Diabetes Prevention Study was only moderate (3%–7%).^33^, ^34^

Noteworthy, in the Diabetes Prevention Study, greater weight loss in the behaviour change treatment group was associated with lower diabetes incidence (58%) compared to the group that received the anti‐diabetes medication metformin (38%) after a median follow‐up of 2.8 years compared to the control group.^33^ These risk reductions showed a sustained legacy effect even at the 15‐year follow‐up recall.^35^ In a meta‐analysis of several diabetes prevention trials, a 5% to 10% weight loss was associated with a significantly reduced type 2 diabetes incidence by 38%.^36^ With more recently developed pharmacotherapies of obesity, greater reduction in type 2 diabetes incidence has been found in association with greater weight loss in individuals treated with liraglutide 3.0 mg once daily or semaglutide 2.4 mg once weekly injections.^37^, ^38^, ^39^

While moderate weight loss in people with obesity and increased type 2 diabetes risk maybe sufficient to prevent or delay the disease, greater weight loss seems to be required to achieve a remission of type 2 diabetes.^40^, ^41^, ^42^ An average weight loss of 7% was not sufficient to significantly improve cardiometabolic endpoints in the Look Ahead trial,^40^ whereas type 2 diabetes remission was related to weight loss >15 kg in the DIRECT trial with a very low‐calorie diet approach and obesity/type 2 diabetes treatment with metabolic surgery in adults and adolescents.^42^, ^43^, ^44^

The examples from type 2 diabetes prevention and treatment trials suggest that the magnitude of weight loss and duration of the disease^45^ represent important determinants of health benefits of weight loss interventions. For other diseases and conditions, the required weight loss to improve or resolve the obesity‐associated complication is variable and depends on the severity of the disorder, but most likely also on the strengths of the causal link between obesity and its specific comorbidity (Figure 3).

## 2 PROGNOSTIC SIGNIFICANCE OF THE OBESITY SUBPHENOTYPE

In clinical practice, it is a frequent observation that human obesity consists of heterogeneous phenotypes. Subtypes of obesity include monogenic, childhood‐onset, syndromic and forms of obesity that vary in the extent how medical, functional and psychological traits are affected.^2^, ^46^, ^47^, ^48^, ^49^, ^50^ A better stratification of obesity subphenotypes may help to prioritise treatment strategies and to identify those people who may benefit the most from weight loss interventions. If the severity of obesity is defined by the number and extent of obesity‐related comorbidities such as in the Edmonton Obesity Staging System, the highest mortality risk can be predicted by the number of comorbid conditions.^26^ In the context of linked electronic health records (1995–2015) in The Health Improvement Network (THIN) including data from more than 3.5 million people in the United Kingdom, the number of obesity complications was also associated with an increasing risk for myocardial infarction or heart failure outcomes.^51^

**FIGURE 4:**
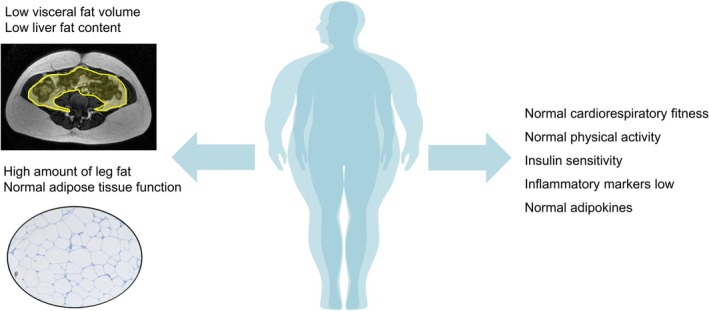
The phenotype of metabolically healthy obesity. Individuals with this subphenotype of obesity are characterised by lower liver and visceral fat, but higher subcutaneous leg fat content, greater cardiorespiratory fitness and physical activity, insulin sensitivity, lower levels of inflammatory markers, and normal adipose tissue function compared to patients with metabolically unhealthy obesity.

On the other hand, there are individuals with obesity that do not develop premature obesity‐related cardiometabolic diseases.^46^ People with metabolically healthy obesity seem to be protected against AT dysfunction^46^, ^48^ and exhibit lower morbidity and mortality risks attributable to obesity.^26^ In order to identify factors underlying metabolically healthy obesity, we recently compared pairs of people with obesity without manifest metabolic comorbidities and cardiovascular risk factors which have been matched for age, sex and BMI into groups of insulin‐sensitive or insulin‐resistant obesity.^48^ Metabolically healthy obesity is characterised by normal fat distribution and preserved AT function, low hepatic fat and normal cardiorespiratory fitness (Figure 4). Interestingly, patients with insulin‐resistant obesity had significantly higher number of macrophages in visceral AT than those with insulin‐sensitive obesity, suggesting that the association between AT inflammation and insulin resistance is not primarily related to fat mass or BMI.^48^ Consistent with our data, another independent study identified visceral AT inflammation as the most significant correlate of metabolically unhealthy obesity.^52^ As another indicator for distinct obesity subtypes, it has been observed that people with obesity who undergo bariatric surgery exhibit different responses with regard to weight loss but also type 2 diabetes and the risk of early weight regain.^53^

Recently, two patterns of human unexplained phenotypic variation in obesity have been proposed by a multi‐dimensional analysis of monozygotic phenotypically discordant twins.^54^ One phenotype is characterised by increased fat mass with only modest reduction of lean mass, the other phenotype exhibits clinical outcomes linked to insulinemia, coordinated increases in fat and lean mass across the body.^54^

Importantly, not every person living with obesity develops typical obesity‐related complications.^3^, ^8^ The heterogeneous association between obesity and its complications despite shared environments maybe explained by genetic and epigenetic factors, but also behavioural patterns that modulate processes and biological pathways involved in the pathogenesis of obesity complications.^8^ We are still at the beginning of understanding the mechanisms defining susceptibility to obesity complications in obesogenic environments, but it is becoming clear that genetic factors and epigenetic mechanisms have an important—and yet to be fully explored—role.^3^, ^55^, ^56^, ^57^ However, there are cardiometabolic diseases (hypertension, type 2 diabetes, fatty liver diseases, gout, dyslipidemia) that typically occur together and are therefore considered as metabolic syndrome.^3^ The parallel occurrence of these diseases suggests a common underlying pathology that includes insulin resistance, abdominal fat distribution and chronic inflammation as potential disease primers.^3^, ^13^, ^14^, ^15^

People with obesity that are characterised by predominantly upper body fat deposition (abdominal subcutaneous and intra‐abdominal visceral AT), intrahepatic, intramyocellular and pancreatic fat are at higher risk of developing type 2 diabetes than those with a lower body, gluteofemoral fat deposition.^46^, ^48^ Predominantly gluteofemoral fat accumulation—a phenotype more frequently observed in premenopausal women—is associated with lower triglyceride and higher HDL‐cholesterol serum concentrations, improved insulin sensitivity, lower fasting blood glucose and insulin concentrations and decreased risk of type 2 diabetes independent of the BMI.^55^

**FIGURE 5:**
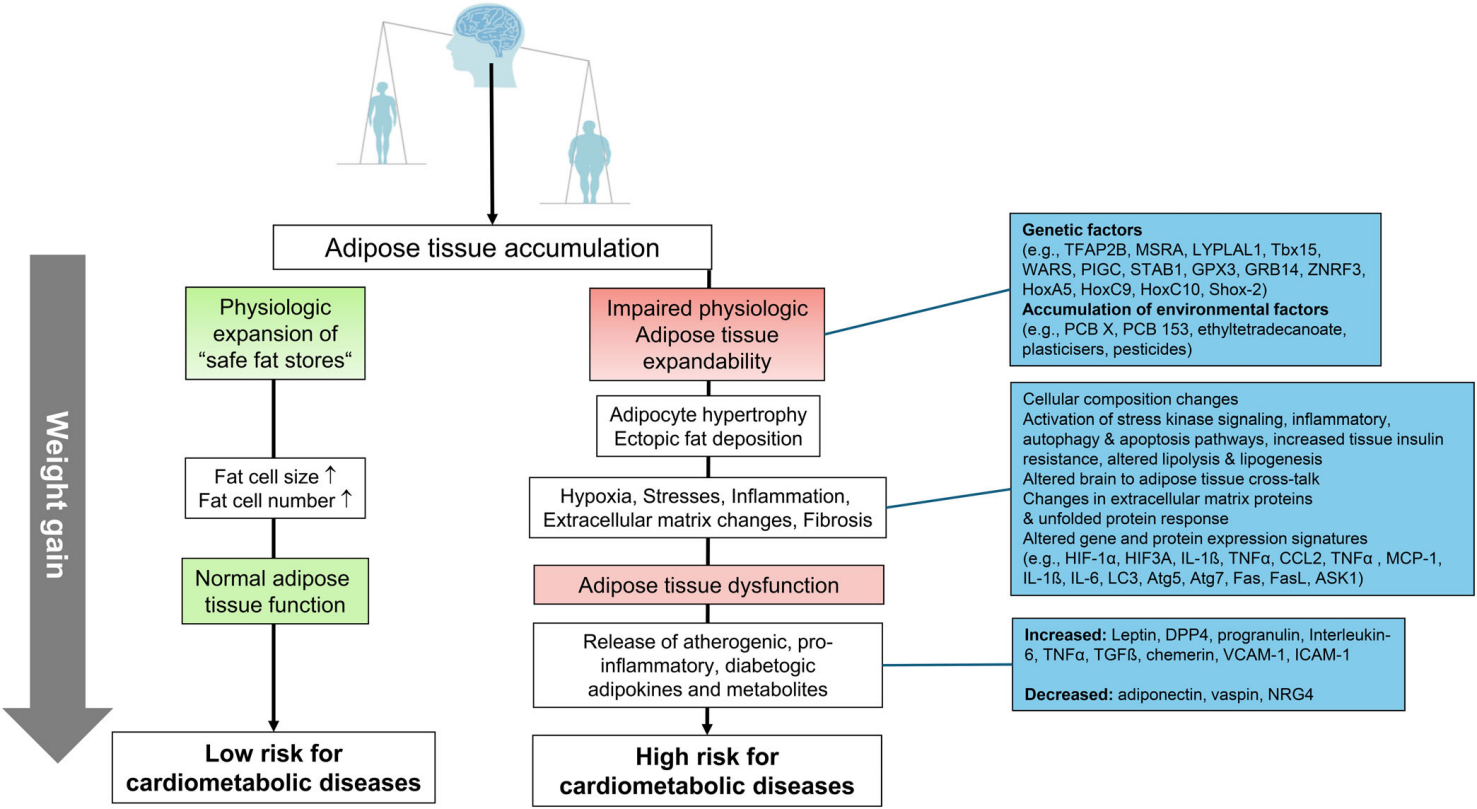
Development of adipose tissue dysfunction. With a chronic positive energy balance and body weight gain, adipose tissue expands due to increased nutrient flux. Under healthy conditions, adipose tissue plasticity allows for an expansion of adipose tissue that is characterised by both hyperplasia and hypertrophy. In the pathogenesis of adipose tissue dysfunction, adipocytes primarily respond to the higher demand for energy storage by increasing their size (adipocyte hypertrophy). Adipocyte hypertrophy contributes to cellular composition changes, activation of stress kinase signalling, inflammatory, autophagy, apoptosis pathways, increased tissue insulin resistance, altered lipolysis and lipogenesis, changes in extracellular matrix proteins and unfolded protein response. These altered pathways are reflected by an altered gene and protein expression signature of dysfunctional adipose tissue (example molecules are shown).

In addition to the health or disease‐promoting aspects of fat deposition, AT dysfunction may contribute to the individual obesity‐related risk to develop cardiometabolic complications.^48^ Although the precise mechanisms of AT dysfunction are not entirely understood, a chronic positive energy balance with weight gain, increased nutrient flux into AT and impaired subcutaneous expandability with adipocyte hypertrophy and local AT insulin resistance and inflammation may initiate a pathology that leads through the release of atherogenic, diabetogenic and pro‐inflammatory signals to damages in target organs (Figure 5). The pathogenesis of AT dysfunction involves alterations in several biological processes and pathways including AT cellular composition changes, activation of stress kinase signalling, inflammatory, autophagy, apoptosis pathways, increased tissue insulin resistance, altered lipolysis and lipogenesis, changes in extracellular matrix proteins, AT fibrosis and unfolded protein response, distinct gene and protein expression signatures, but also alterations in the brain to AT signalling (Figure 5).^21^, ^46^, ^48^, ^56^, ^57^, ^58^, ^59^ Importantly, AT dysfunction may be initiated by impaired AT plasticity in response to metabolic cues, which contribute to the development of cardiometabolic diseases.^58^ It has been suggested that the capacity for healthy or unhealthy AT remodelling may depend on the intrinsic diversity of adipose progenitor cells.^58^ Moreover, the central nervous system may play an additional role in the development of impaired AT function through alterations of the brain‐to‐periphery cross‐talk. The brain communicates with AT through sensory and sympathetic nerves that modulate metabolic processes.^59^

## 3 ALTERED SIGNALS FROM AT MAY LINK OBESITY TO COMPLICATIONS

After the discovery that AT is an active endocrine organ,^60^, ^61^, ^62^ it became clear that adipokines play specific roles in the regulation of appetite and satiety, immune response and inflammation, glucose and lipid metabolism, insulin sensitivity, hypertension, vascular growth and function, atherosclerosis development, bone development, growth and other biological processes.^59^, ^60^, ^61^, ^62^, ^63^, ^64^, ^65^, ^66^ Altered secretion of adipokines (e.g., leptin, adiponectin), cytokines (e.g., monocyte chemoattractant protein‐1 [MCP‐1], chemerin, interleukin‐6), metabolites (fatty acids), exosomes,^67^ immune cells and others are symptoms of AT dysfunction. An adverse, diabetogenic, pro‐inflammatory and atherogenic adipokine secretion pattern links obesity and AT dysfunction (including conditions like lipodystrophy) to obesity‐related cardiometabolic diseases.^65^ Therefore, adipokines are considered both as biomarkers for AT dysfunction and as tools for the prevention and treatment of obesity‐related diseases.^64^, ^65^ Changes in adipokine production under conditions of AT dysfunction dramatically change AT communication with organs including the brain, pancreas, liver, skeletal muscle, heart, vasculature, immune system and distant fat depots.^64^, ^65^, ^68^ Indeed, adipokines regulate physiological functions of several organs and in states of AT dysfunction adipokines have disease‐modifying effects contributing to end‐organ damage and pathological outcomes (Table 1).

**TABLE 1:** Potential mechanisms linking obesity to comorbidities.

Obesity‐related mechanism	Resulting condition	References
Insulin resistance β‐cell dysfunction Ectopic fat deposition Diabetogenic signals from AT	Type 2 diabetes	^4^, ^41^, ^63^
Increased sympathetic activity Hyperaldosteronism Altered natriuresis (visceral fat distribution) OSAS	Hypertension	^69^, ^70^
Increased lipids release from AT Ectopic fat deposition Chronic systemic inflammation Genetic factors Altered bile acid composition	MAFLD	^4^, ^71^, ^72^
Insulin resistance Hyperinsulinemia	Dyslipidemia	^73^, ^74^
Mechanical factors Central nervous system mechanisms	Obstructive sleep apnea	^4^, ^69^
Hypertension, dyslipidemia, insulin resistance, hyperglycaemia, inflammation, atherogenic adipokine profile, OSAS	Vascular diseases (Myocardial infarction, stroke, peripheral artery disease)	^4^, ^5^, ^69^, ^70^
Mechanical	Gastroesophageal reflux Incontinence Osteoarthritis Low cardiorespiratory fitness	^4^, ^32^, ^75^
Social stigmatisation Central nervous system mechanisms Altered reward, addictive behaviour	Mental health disorders	^4^
Insulin resistance Altered sex hormones Adipokines	Impaired fertility	^64^, ^65^, ^76^
Adipokines and incretin hormones	Altered appetite and satiety	^64^, ^65^, ^76^
Altered pro‐coagulatory signals mechanical	Thrombosis, pulmonary embolism	^60^, ^64^, ^65^, ^68^
Hypertension, OSAS Inflammation Activation of sympathetic nervous system Increased plasma volume Increased lipid flux, insulin resistance	Chronic heart failure	^4^, ^77^

Abbreviations: MAFLD, metabolic dysfunction‐associated fatty liver disease; OSAS, obstructive sleep apnea syndrome.

*Source*: The table has been modified from ^4^.

Altered adipokine secretion upon AT dysfunction may affect the periphery‐brain cross‐talk. For example, leptin regulates appetite, satiety and locomotor activity (Table 1). AT dysfunction and fatty liver diseases are closely interconnected. Adipokine secretion and changes in metabolite release may contribute to promote fat accumulation in the liver and liver insulin resistance.^66^ As an example, leptin may improve hepatic steatosis via indirect effects and directly via hepatic activation of adenosine monophosphate‐activated protein kinase (AMPK).^68^ Adiponectin affects liver metabolism through its receptors and suppresses hepatic glucose output via phosphorylation of AMPK in addition to lowering hepatic pro‐inflammatory ceramides and inhibiting the fibrogenic effects of transforming growth factor‐β.^78^

Taken together, altered cross‐talk between AT and organs affected by obesity complications are candidates for future pharmacological treatment strategies.^76^ Metreleptin is already used as a pharmacotherapy in individuals with congenital leptin deficiency and lipodystrophies.^76^

Given the important role of AT and altered inter‐organ cross‐talk as pathomechanisms for obesity‐related complications, the hypothesis was tested that reducing AT by liposuction or omentectomy may improve metabolic traits in people with obesity.^47^, ^79^ However, in contrast to weight loss achieved by negative energy balance (bariatric surgery, low‐calorie diets, pharmacotherapy), surgical removal of AT does not result in metabolic benefits.^47^, ^79^ Even the removal of 10 kg of abdominal subcutaneous AT by liposuction (equal to 20% reduction in total body fat mass) did not affect insulin sensitivity or parameters of glucose and lipid metabolism in women with obesity with or without type 2 diabetes.^47^ Moreover, a laparoscopic omentectomy removing a third of intra‐abdominal AT did not have beneficial effects on insulin resistance in people with type 2 diabetes.^79^ These data raise the hypothesis that obesity treatment strategies inducing a negative energy balance rather than reducing fat mass by surgical approaches are required to achieve the metabolic benefits observed in real world and randomised controlled weight‐loss trial settings.^47^

## 4 CLINICALLY MOST RELEVANT OBESITY‐RELATED COMPLICATIONS

The Global Burden of Disease Obesity Collaborators 2015 evaluated the health effects of overweight and obesity in 195 Countries over 25 Years with regard to high BMI‐related mortality and health outcomes.^5^ In 2015, high BMI contributed to 4.0 million deaths (95% uncertainty interval, 2.7–5.3), which represented 7.1% (95% uncertainty interval, 4.9–9.6) of the deaths from any cause.^5^ Cardiovascular diseases were with 41% of the leading cause of mortality attributable to high BMI.^5^ Diabetes was the second leading cause of death in 2015 followed by chronic kidney disease and cancer.^5^ Diabetes and musculoskeletal disorders were among the leading causes for years lived with disabilities.^5^ Moreover, a pooled cohort analysis involving 1.8 million participants showed that nearly half the excess risk for ischemic heart disease and more than 75% of the excess risk for stroke that was related to high BMI were mediated through a combination of raised levels of blood pressure, total serum cholesterol, and fasting plasma glucose.^79^

**FIGURE 6:**
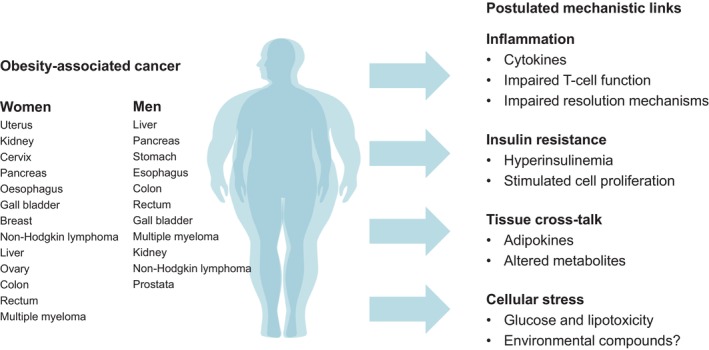
Obesity and cancer. Obesity is associated with an increased risk to develop specific types of cancer with differences between men and women.^5^, ^67^ Proposed mechanisms linking obesity and adipose tissue dysfunction to increased risk to obesity‐related cancers.^68^

Certain obesity‐related cancers also significantly contribute to mortality associated with high BMI. The systematic evaluation of prospective observational studies provided data supporting a causal relationship between obesity and cancers of the oesophagus, colon and rectum, liver, gallbladder and biliary tract, pancreas, breast, uterus, ovary, kidney and thyroid.^5^ Other systematic studies reported partly overlapping and additional distinct types of cancer that are associated with obesity and increase the obesity‐related health burden (Figure 6).^80^, ^81^, ^82^

Together, the data on obesity‐related health burden, their effects on increased mortality and years lived with disability strongly suggest that clinical obesity treatment should focus on reducing the underlying rate of cardiovascular diseases, type 2 diabetes and cancer, because it could substantially reduce the burden of disease related to high BMI.^5^

An early access to effective weight loss therapies and long‐term obesity management strategies treatment including behavioural, pharmacological and/or surgical therapy should become a public health priority.^4^, ^75^ Given the high number of obesity‐related complications, the more in‐depth discussion below focusses on the obesity‐related diseases that are most relevant in the clinical setting, because of their association with increased mortality and impaired quality of life.

## 5 WHAT IS A CLINICALLY MEANINGFUL WEIGHT LOSS TO IMPROVE OBESITY COMPLICATIONS?

Despite the recognition of obesity as a disease and existing clinical practice guidelines, there is a large unmet need for a patient‐centred, evidence‐based obesity treatment from both primary care providers, endocrinologists and other specialists.^75^, ^83^ There are still several obstacles to translate current knowledge about the causes of obesity and modern treatment options for people living with obesity. Such barriers to initiating obesity treatment in primary care include a perceived lack of time during office visits, the fact that obesity treatment is not prioritised over other health concerns, and the stigma that patients with obesity are not sufficiently motivated to lose weight or uncomfortable discussing their weight.^75^, ^84^ In addition, the lack of training provided by most medical schools, but also in postgraduate programmes and for other healthcare professionals frequently leads to insufficient skills to initiate and support obesity management.^75^, ^85^, ^86^, ^87^

After overcoming these barriers in obesity treatment, the question remains, what are realistic and meaningful weight loss targets. In 2007, the US Food and Drug Administration (FDA) defined a weight loss of ≥5% as a reasonable marker to evaluate the efficacy of anti‐obesity medications.^88^ This ‘efficacy target’ is mainly derived from clinical trial data providing evidence that 5% and more weight loss provides sufficient health benefits.^89^, ^90^ Therefore, many practice guidelines for the management of obesity adopted the ≥5% weight loss target.^91^ However, the most recent recommendations for the standard of care by the American Diabetes Association (ADA) note that a sustained loss of >10% of body weight usually confers greater benefits, including disease‐modifying effects and possible remission of type 2 diabetes, and may improve long‐term cardiovascular outcomes and mortality.^91^

This statement is in line with the notion that there is a continuum of clinically meaningful weight loss, which varies among individuals and depends on specific obesity subtypes, severity of disease, complications and others. The recommendation from the ADA is based on semaglutide and tirzepatide trials in people with type 2 diabetes demonstrating that >10% is realistic and may lead to greater health benefits compared to lower weight loss targets.^92^, ^93^, ^94^ Whereas a sustained weight loss of as little as 2–5% has shown significant benefits in cardiovascular risk factors,^95^ for many obesity‐related complications, clinically relevant improvements require greater weight loss (Figure 3).

Obesity management should reinforce the concept that clinically meaningful weight loss cannot be based on weight loss alone and requires a patient‐individualised approach focusing on additional improvements in quality of life, health outcomes, and reducing complications.^24^, ^75^

## 6 CARDIOVASCULAR DISEASES

Cardiovascular diseases represent the main driver of premature mortality in people with obesity.^5^ Obesity increases the risk for cardiovascular diseases both indirectly through adverse effects on risk factors including hypertension, type 2 diabetes, and dyslipidemia^4^, ^5^ and may additionally directly alter vascular function although the exact mechanisms are not fully understood (Table 1).

### 6.1 Hypertension

Obesity increases the risk to develop essential hypertension by about 70% through several mechanisms (Table 1).^4^ Effects of increased visceral fat mass on natriuresis, activation of the sympathetic nervous system (potentially initiated by leptin activation), activation of the renin‐aldosterone action, higher AT secretion of vasoactive substances, and obstructive sleep apnea may causally link obesity and impaired AT function to hypertension.^69^, ^70^ Interventions that reduce body weight have been shown to improve or resolve hypertension associated with obesity. This effect is related to the extent of weight loss and has been shown for behaviour (LOOK AHEAD best responders^96^), pharmacological (e.g., liraglutide,^97^ dulaglutide,^98^ semaglutide,^38^ tirzepatide^99^) and surgical interventions.^100^

### 6.2 Atherosclerotic vascular diseases

In epidemiological studies, obesity was shown to be associated with an increased risk of coronary artery disease, stroke and cardiovascular mortality even in the absence of overt cardiometabolic risk factors.^5^, ^16^, ^51^, ^101^, ^102^, ^103^, ^104^ Factors associated with abdominal fat distribution may be important drivers of the increased cardiovascular disease risk in obesity and parameters of central obesity are stronger predictors of cardiovascular mortality than BMI.^16^

The link between obesity and increased cardiovascular disease risk might be causally mediated by genetic risk factors for adverse fat distribution as determined by the waist‐to‐hip ratio or insulin resistance in genome‐wide association studies.^105^, ^106^

The obvious approach to reduce the obesity‐associated risk for atherosclerotic vascular diseases is caloric restriction in combination with increasing physical activity.^107^ A nutritional energy deficit can be achieved by specific strategies including portion size control, reduction or elimination of ultra‐processed foods such as sugar‐sweetened beverages, reduction of alcohol consumption, and increased fruit and vegetable intake. In short‐term trials, ~5%–10% weight reduction has been demonstrated for low fat‐vegan, vegetarian style, low carbohydrate and Mediterranean diets.^108^

According to a meta‐analysis comparing dietary macronutrient patterns and dietary programmes for weight and CV risk factor reduction in overweight and obese adults, most dietary patterns resulted in similar, only modest short‐term weight loss, but with substantial improvements in cardiovascular risk factors. However, there is no evidence that caloric restriction alone or in combination with increasing physical activity significantly reduces cardiovascular (CV) events. As an example, in the Look AHEAD trial, 5145 patients with overweight/obesity and type 2 diabetes were randomised to an intensive lifestyle intervention (weight loss promotion through reduced caloric intake and increased physical activity) or regular care.^40^ Despite greater weight loss (−8.6% vs. −0.7%), and greater reduction in HbA1c and blood pressure levels, the intensive lifestyle intervention did not reduce the rate of CV events.^40^ However, a *post‐hoc* analysis revealed a significant reduction in CV events in patients achieving ≥10% weight loss in the first year of intervention.^96^

Until recently, there was no formal evidence that weight loss interventions in people with obesity without type 2 diabetes may significantly decrease cardiovascular endpoints. Data from bariatric surgery indicated that the extent of weight loss may be associated with reduced risk for cardiovascular endpoints.^31^, ^109^ But this observation has not been tested in a randomised controlled trial.

The SELECT trial was the first prospective randomised controlled trial that demonstrated that in patients with pre‐existing cardiovascular disease and overweight or obesity but without diabetes, a significant weight reduction achieved by weekly subcutaneous semaglutide at a dose of 2.4 mg was superior to placebo in reducing the incidence of death from cardiovascular causes, nonfatal myocardial infarction, or nonfatal stroke at a mean follow‐up of 39.8 months.^92^, ^109^ In a total of 17 604 participants, the primary cardiovascular end‐point event occurred in 569 of the 8803 patients (6.5%) in the semaglutide group and in 701 of the 8801 patients (8.0%) in the placebo group (hazard ratio, 0.80; 95% confidence interval, 0.72–0.90; *p* <0.001).^92^ However, the beneficial cardiovascular outcome effects of the SELECT trial cannot be entirely explained by weight loss effects of semaglutide.^110^ Participants of the SELECT trial who experienced only modest weight loss with semaglutide also had cardiovascular benefits.^92^ Moreover, in type 2 diabetes cardiovascular outcomes trials for liraglutide^97^ and dulaglutide,^98^ a modest weight loss was associated with significant benefits on cardiovascular outcome endpoints.

### 6.3 Heart failure

Obesity is a risk factor for heart failure even if disease risk was adjusted for type 2 diabetes, hypertension, age, smoking, physical activity and dyslipidemia as shown in the Atherosclerosis Risk in Communities (ARIC) study.^77^ A BMI >35 kg/m^2^ was associated with a 3.7‐fold increased risk of heart failure. The pathogenic factors linking obesity to heart failure are multiple and include direct adiposity‐related and indirect mechanisms (Table 1).

For example, increased plasma volume contributes to high‐output cardiac failure and metabolic factors predisposing to heart failure with predominantly diastolic dysfunction including cardiac steatosis, insulin resistance, altered substrate use from glucose to fat.^4^

Recently, the STEP‐HFpEF trials demonstrated that semaglutide treatment improves heart failure‐related symptoms and physical limitations and reduces body weight in participants with obesity‐related heart failure with preserved ejection fraction.^111^ In the SUMMIT trial, compared to placebo treatment with tirzepatide led to a 38% lower relative risk for an endpoint composed of death from cardiovascular causes or worsening of heart failure in patients with heart failure with preserved ejection fraction and obesity.^112^

### 6.4 Atrial fibrillation

Atrial fibrillation is the most frequently associated arrhythmia in people with obesity accounting for approximately 20% of the cases.^4^ Among the pathogenic factors (Table 1), obstructive sleep apnea, secretion of pro‐inflammatory factors from epicardial and visceral fat depots may lead to left atrial remodelling and atrial fibrillation.^113^ Atherosclerotic coronary disease may further increase the obesity‐related risk for atrial fibrillation.^4^ A reduced burden of atrial fibrillation has been shown in patients with obesity after bariatric surgery.^114^ These beneficial effects of surgical obesity treatment have been attributed to reductions in inflammation, left atrial and ventricular remodelling, OSAS, blood pressure, and improved glycemic control beyond the weight loss effects.^113^, ^114^

## 7 METABOLIC DISEASES

Metabolic diseases including type 2 diabetes, dyslipidemia and metabolic dysfunction‐associated fatty liver disease (MAFLD) belong to the most common obesity‐related complications.^5^ The mechanisms that promote obesity‐related metabolic complications are multifactorial and include insulin resistance, adverse fat distribution, altered adipokine secretion, subclinical chronic metabolic inflammation and impaired AT dysfunction (Table 1). Recently, it has been shown that high adiponectin serum concentrations (reflecting normal AT function) were associated with the highest likelihood for ageing free of major chronic diseases.^115^ Targeting metabolic inflammation may become a future strategy to prevent or treat cardiometabolic and malign obesity complications. AT inflammation appears to be an important hallmark of metabolic inflammation,^48^ but so far no specific treatment exists to reverse AT inflammation. Glucose and lipid‐lowering medications may exert anti‐inflammatory effects and may therefore contribute to improve cardiometabolic obesity complications.^116^ More specifically targeting metabolic inflammation in obesity, the development of cell‐based immunotherapies including lymphocyte‐based immunotherapies (e.g., rituximab that targets CD20 to delete B cells; alemtuzumab that targets CD52 to deplete lymphocytes; foralumab that targets CD3 to delete T cells) advanced recently.^116^ In addition, clinical trials with anti‐inflammatory drugs such as salsalate, anakinra, canakinumab medications targeting TNFα, CCX140‐B and others suggest that targeting inflammation may become a strategy to improve cardiometabolic obesity complications by targeting one of the key mechanisms linking AT dysfunction to end‐organ damage.^116^

### 7.1 Type 2 diabetes

The obesity‐attributable risk of type 2 diabetes may increase up to >10‐fold with increasing BMI (Figure 2). Obesity and AT dysfunction have a negative impact on the key pathogenetic factors for the development of type 2 diabetes: impaired insulin sensitivity and insulin secretion, hepatic steatosis and insulin resistance, increased diabetogenic signals from AT (adipokines, lipids, pro‐inflammatory cytokines), reduced diversity of gut microbiota and impaired skeletal muscle insulin sensitivity.^4^, ^9^, ^10^, ^64^, ^65^, ^66^, ^117^, ^118^ Weight loss is a main target in the treatment of patients with type 2 diabetes^90^ and can not only prevent diabetes in people with prediabetes,^33^, ^34^ but also lead to sustained remission of the disease after intensive dietary interventions, such as the DiRECT study,^41^ or bariatric surgery.^100^, ^117^, ^118^ Higher likelihood of type 2 diabetes remission upon weight loss is related to an early intervention, shorter duration of the disease, younger age and the extent of weight loss.^119^, ^120^ In the state of prediabetes, behavioural interventions that led to an average weight loss of 3 kg significantly reduced the incidence of type 2 diabetes.^33^, ^34^ However, a greater weight loss of >10% seems to be required to reverse obesity‐related type 2 diabetes as behavioural, pharmacotherapy and obesity surgery interventions suggest.^38^, ^41^, ^92^, ^93^, ^94^, ^99^, ^119^ Incretin‐based pharmacotherapies, particularly with semaglutide and tirzepatide, have the potential to change the disease trajectory of type 2 diabetes and may even lead to the remission of the disease.^27^, ^28^, ^38^, ^92^, ^94^, ^99^ Importantly, the extent of beneficial effects achieved by incretin‐based therapies does not seem to be only attributable to weight loss.

Sodium‐glucose cotransporter‐2 (SGLT2) inhibitors are now widely used in the treatment of patients with type 2 diabetes, chronic kidney disease and heart failure.^121^, ^122^, ^123^ SGLT2 inhibitor treatment has beneficial effects of several obesity complications that are not entirely explained by their weight loss effect. SGLT2 inhibitors demonstrated great benefits for heart failure outcomes and improve blood glucose parameters, blood pressure, body weight and liver function.^121^, ^122^, ^123^ Although SGLT2 inhibitors are not approved for the treatment of obesity, they can be used to treat important obesity complications specifically in patients with type 2 diabetes, HFpEF or HFrEF and chronic kidney disease.

However, the effects of incretin‐based therapies and SGLT2 inhibitors beyond weight loss and improved glycemic parameters need to be further explored.

### 7.2 Dyslipidemia

Dyslipidemia in people with obesity is typically characterised by elevated fasting serum triglycerides, low HDL‐cholesterol and increased small‐dense LDL‐cholesterol levels.^73^

In the context of the metabolic syndrome, these alterations in lipid parameters maybe considered a disease primer for obesity‐related cardiometabolic diseases.^73^ Obesity‐related systemic and hepatic insulin resistance as well as hyperinsulinemia are the most relevant mechanisms underlying the epidemiological associations of obesity dyslipidemia beyond the association with increasing BMI.^5^ However, obesity is not causally related to the well‐established cardiovascular risk factor LDL‐hypercholesterinemia. Particularly in patients with obesity and insulin resistance, substantial weight loss by bariatric surgery or tirzepatide pharmacotherapy had beneficial effects on obesity‐related dyslipidemia.^71^, ^74^

### 7.3 Metabolic dysfunction‐associated fatty liver disease

MAFLD is associated with obesity and visceral fat distribution and increases the risk to develop into more advanced liver disease and hepatocellular carcinoma.^72^ The prevalence of MAFLD has increased in parallel with the obesity pandemic and affects more than a fourth of the adult population in Western countries. MAFLD is projected to be the leading cause of liver cirrhosis and an indication for liver transplant therapy.^72^, ^124^ Obesity and MAFLD may be linked by insulin resistance, visceral fat accumulation and the release of adverse metabolite and adipokine signals.^71^ In addition to hypercaloric nutrition, high fructose intake may promote fatty liver diseases independently of the degree of adiposity or BMI.^125^

Behavioural interventions that involve caloric restriction or reduction in carbohydrate intake can effectively improve MAFLD.^126^, ^127^ A weight loss of greater than 10% has been shown to reduce or reverse hepatic steatosis and metabolic dysfunction‐associated steatohepatitis (MASH). Improved MASLD and MASH outcome parameters have been shown for weight loss achieved by hypocaloric diets,^126^, ^127^ obesity pharmacotherapies,^128^, ^129^, ^130^ and bariatric surgery.^131^ Whether obesity pharmacotherapies are also effectively improving MAFLD in more advanced diseases states is currently under investigation. The ‘Effect of Semaglutide in Subjects with Non‐cirrhotic Non‐alcoholic Steatohepatitis' (ESSENCE)’ trial is currently undergoing and aims to evaluate the effect of subcutaneous semaglutide 2.4 mg in participants with biopsy‐proven MASH and fibrosis stages 2 or 3.^132^ The two primary endpoints of the ESSENCE trial are (i) resolution of steatohepatitis and no worsening of liver fibrosis and (ii) improvement in liver fibrosis and no worsening of steatohepatitis.^132^

## 8 CANCER

Obesity‐related cancers contribute to reduced life expectancy in people living with obesity.^5^ Epidemiological data suggest a significantly increased obesity‐related risk for oesophagus, colon and rectum, liver, gallbladder and biliary tract, pancreas, breast, uterus, ovary, kidney, thyroid and other cancers (Figure 6).^5^, ^81^, ^82^ The risk to develop cancer later in life seems to be partly related to childhood and adolescent obesity.^4^ In addition to the increased risk to develop certain types of cancer, obesity is associated with worse outcomes after cancer therapies.^129^ Several mechanisms have been proposed to underlie the association of obesity to specific cancer risks in addition to the common soil hypothesis that overeating and low physical activity may in parallel promote both obesity and cancer (Figure 6). Obesity is frequently associated with insulin resistance and hyperinsulinemia that may via mitogenic effects contribute to the cancer pathogenesis.^81^, ^82^ Moreover, chronic subclinical inflammation in obesity, cellular stress upon nutrient excess, glucose and lipid toxicity and altered AT cross‐talk may relate obesity to some cancer types (Figure 6). Importantly, these pathomechanisms are not necessarily related to increased body mass or higher BMI. Recently, obesity was shown to be associated with specific driver mutations in lung adenocarcinoma, endometrial carcinoma and cancers of unknown primaries, independent of clinical covariates, demographic factors and genetic ancestry.^133^, ^134^ Obesity may therefore be considered a driver of etiological heterogeneity in some cancers.^134^

Weight loss may reduce the obesity‐related cancer risk. There is supporting evidence from the Women's Health Initiative observational study, suggesting that intentional weight loss of >5% protects against 11 types of cancer in women.^135^ In observations of patients after bariatric surgery, weight loss reduced the incidence of breast and gynaecological malignancies as well as overall cancer mortality.^136^, ^137^

Obesity increases the risk to develop distinct types of cancer most likely by metabolic abnormalities and inflammation. Whether weight loss strategies can reduce the incidence of obesity‐related cancers needs to be systematically investigated in prospective studies.

## 9 CONCLUSIONS AND PERSPECTIVES

Obesity is a chronic progressive multisystem disease that can lead to adverse medical, functional and mental health outcomes ultimately reducing life expectancy. Obesity cannot be cured, but increasingly better treated. The definition of obesity based on BMI only does not sufficiently reflect the individual disease burden and obesity complications. Therefore, the EASO^2^ and the Lancet Commission on clinical obesity^8^ proposed new diagnostic criteria that include direct measurement of body fat or at least one anthropometric criterion (e.g., waist circumference, waist‐to‐hip ratio, waist‐to‐height ratio) in addition to BMI to define excess adiposity. Whether these diagnostic criteria and a proposed distinction into preclinical and clinical obesity will be practicable and lead to a better prevention or treatment of obesity complications remains open and should be tested longitudinally in clinical settings.

The ultimate goal of obesity management should not only be weight reduction but improving the clinical manifestations of obesity and prevent progression to end‐organ damage. To achieve that, we need better criteria to prioritise treatment in people with (clinical) obesity that benefit the most from receiving timely, evidence‐based treatment.

Timely and structured assessments and interventions including behavioural, pharmacological and surgical treatment strategies can prevent or reverse obesity complications. In subclinical or early disease states, moderate weight loss of 3%–5% seems to be sufficient to prevent the manifestation of type 2 diabetes and other cardiometabolic diseases. However, once complications have developed, a greater weight loss of >10% may be required to significantly improve obesity‐associated disorders or lead to the remission of adverse health outcomes.

In this context, novel incretin‐based therapies have revolutionised the treatment of people living with obesity and type 2 diabetes. With semaglutide and tirzepatide, average weight loss of >15% or >20%, respectively, could be achieved in people with obesity and for people with type 2 diabetes incretin‐based therapies provide impressive weight loss and glucose parameter improvements in clinical trials.^27^, ^28^, ^38^, ^92^, ^94^, ^99^, ^138^ Moreover, the landscape of obesity complication management including MAFLD, heart failure, chronic kidney disease, obstructive sleep apnea and others will be changed by current and future incretin‐based therapies.^27^ In the future, medications like GLP‐1 receptor agonists, dual incretin receptor agonists, combination therapies (e.g., cagrilintide/semaglutide) or triple agonists (e.g., retatrutide) have the potential to narrow the efficacy gap between pharmacotherapy and obesity surgery.^27^

While short‐term efficacy and safety of behavioural and pharmacological obesity treatment have been demonstrated, there is a need to evaluate the long‐term effects and sustainability of these treatments. This particularly applies to obesity pharmacotherapy. Future studies need to address long‐term patient outcomes under real‐world conditions, identify mechanisms of non‐ versus very good response to pharmacotherapies, and investigate long‐term safety and sustainability of weight loss similar to long‐term health monitoring after bariatric surgery.

Weight loss interventions target key mechanisms linking obesity to its complications including insulin resistance, impaired adipokine secretion with higher pro‐inflammatory adipokines (e.g., MCP‐1, progranulin, chemerin), altered AT‐brain cross‐talk (e.g., high leptin levels in obesity) and lower secretion of cardiometabolically ‘protective’ adipokines (e.g., adiponectin), visceral fat deposition and AT inflammation. However, to improve or remit obesity complications, an early and effective therapy is required, because the treatment success depends on the extent of weight loss and a shorter disease duration. People living with obesity should therefore have easy access to structured obesity management programmes. Unfortunately, there are still many barriers in healthcare systems resulting in insufficient structures and limited access to guideline‐driven obesity management. Reducing stigma and societal attitudes are important obstacles that need to be overcome. Although recent data from real‐world studies and randomised controlled obesity management trials provide guidance for a state‐of‐the‐art obesity treatment, we still need to better understand and target the causal factors that underlie obesity complications. The ultimate goal is to improve quality of life and life expectancy for people with obesity.

## CONFLICT OF INTEREST STATEMENT

MB received honoraria as a consultant and speaker from Amgen, AstraZeneca, Bayer, Boehringer‐Ingelheim, Daiichi‐Sankyo, Lilly, Novo Nordisk, Novartis, Pfizer and Sanofi.

### PEER REVIEW

The peer review history for this article is available at https://www.webofscience.com/api/gateway/wos/peer-review/10.1111/dom.16263.

## References

[1] WHO . World Health Organization. 2024 Accessed October 30, 2024. www.who.int/mediacentre/factsheets/fs311/en/

[2] Busetto L , Dicker D , Frühbeck G , et al. A new framework for the diagnosis, staging and management of obesity in adults. Nat Med. 2024;30(9):2395‐2399. 38969880 10.1038/s41591-024-03095-3

[3] Blüher M . Obesity: global epidemiology and pathogenesis. Nat Rev Endocrinol. 2019;15:288‐298. 30814686 10.1038/s41574-019-0176-8

[4] Sarma S , Sockalingam S , Dash S . Obesity as a multisystem disease: trends in obesity rates and obesity‐related complications. Diabetes Obes Metab. 2021;23(Suppl. 1):3‐16. 10.1111/dom.14290 33621415

[5] GBD 2015 Obesity Collaborators , Afshin A , Forouzanfar MH , et al. Health effects of overweight and obesity in 195 countries over 25 years. N Engl J Med. 2017;377(1):13‐27. 28604169 10.1056/NEJMoa1614362 PMC5477817

[6] Tremmel M , Gerdtham UG , Nilsson PM , Saha S . Economic burden of obesity: a systematic literature review. Int J Environ Res Public Health. 2017;14(4):435. 28422077 10.3390/ijerph14040435 PMC5409636

[7] Ling J , Chen S , Zahry NR , Kao TA . Economic burden of childhood overweight and obesity: a systematic review and meta‐analysis. Obes Rev. 2023;24(2):e13535. 36437105 10.1111/obr.13535 PMC10078467

[8] Rubino F , Cummings DE , Eckel RH , et al. Definition and diagnostic criteria of clinical obesity. Lancet Diabetes Endocrinol. 2025;S2213‐8587(24):00316‐4. 10.1016/S2213-8587(24)00316-4 PMC11870235 39824205

[9] Kim KK , Haam JH , Kim BT , et al. Evaluation and treatment of obesity and its comorbidities: 2022 update of clinical practice guidelines for obesity by the Korean Society for the Study of obesity. J Obes Metab Syndr. 2023;32(1):1‐24. 36945077 10.7570/jomes23016 PMC10088549

[10] Rodbard HW , Bays HE , Gavin JR 3rd , et al. Rate and risk predictors for development of self‐reported type‐2 diabetes mellitus over a 5‐year period: the SHIELD study. Int J Clin Pract. 2012;66(7):684‐691. 22698420 10.1111/j.1742-1241.2012.02952.x

[11] Must A , Spadano J , Coakley EH , Field AE , Colditz G , Dietz WH . The disease burden associated with overweight and obesity. JAMA. 1999;282(16):1523‐1529. 10546691 10.1001/jama.282.16.1523

[12] GBD 2021 Diabetes Collaborators . Global, regional, and national burden of diabetes from 1990 to 2021, with projections of prevalence to 2050: a systematic analysis for the global burden of disease study 2021. Lancet. 2023;402(10397):203‐234. 37356446 10.1016/S0140-6736(23)01301-6 PMC10364581

[13] Stefan N , Schulze MB . Metabolic health and cardiometabolic risk clusters: implications for prediction, prevention, and treatment. Lancet Diabetes Endocrinol. 2023;11(6):426‐440. 37156256 10.1016/S2213-8587(23)00086-4

[14] Lotta LA , Wittemans LBL , Zuber V , et al. Association of genetic variants related to gluteofemoral vs abdominal fat distribution with type 2 diabetes, coronary disease, and cardiovascular risk factors. JAMA. 2018;320(24):2553‐2563. 30575882 10.1001/jama.2018.19329 PMC6583513

[15] Lotta LA , Gulati P , Day FR , et al. Integrative genomic analysis implicates limited peripheral adipose storage capacity in the pathogenesis of human insulin resistance. Nat Genet. 2017;49(1):17‐26. 27841877 10.1038/ng.3714 PMC5774584

[16] Pischon T , Boeing H , Hoffmann K , et al. General and abdominal adiposity and risk of death in Europe. N Engl J Med. 2008;359(20):2105‐2120. 19005195 10.1056/NEJMoa0801891

[17] Neeland IJ , Ross R , Després JP , et al. International atherosclerosis society; international chair on cardiometabolic risk working group on visceral obesity. Visceral and ectopic fat, atherosclerosis, and cardiometabolic disease: a position statement. Lancet Diabetes Endocrinol. 2019;9:715‐725. 10.1016/S2213-8587(19)30084-1 31301983

[18] NCD Risk Factor Collaboration (NCD‐RisC) . Worldwide trends in underweight and obesity from 1990 to 2022: a pooled analysis of 3663 population‐representative studies with 222 million children, adolescents, and adults. Lancet. 2024;403(10431):1027‐1050. 38432237 10.1016/S0140-6736(23)02750-2 PMC7615769

[19] Gujral UP , Pradeepa R , Weber MB , Narayan KM , Mohan V . Type 2 diabetes in south Asians: similarities and differences with white Caucasian and other populations. Ann N Y Acad Sci. 2013;1281(1):51‐63. 23317344 10.1111/j.1749-6632.2012.06838.x PMC3715105

[20] Abate N , Chandalia M . Risk of obesity‐related cardiometabolic complications in special populations: a crisis in Asians. Gastroenterology. 2017;152(7):1647‐1655. 28192110 10.1053/j.gastro.2017.01.046

[21] Goossens GH . The metabolic phenotype in obesity: fat mass, body fat distribution, and adipose tissue function. Obes Facts. 2017;10(3):207‐215. 28564650 10.1159/000471488 PMC5644968

[22] Ashwell M , Gunn P , Gibson S . Waist‐to‐height ratio is a better screening tool than waist circumference and BMI for adult cardiometabolic risk factors: systematic review and meta‐analysis. Obes Rev. 2012;13(3):275‐286. 22106927 10.1111/j.1467-789X.2011.00952.x

[23] Garvey WT , Mechanick JI , Brett EM , et al. American Association of Clinical Endocrinologists and American College of Endocrinology comprehensive clinical practice guidelines for medical care of patients with obesity. Endocr Pract. 2016;22(Suppl 3):1‐203. 10.4158/EP161365.GL 27219496

[24] Wharton S , Lau DCW , Vallis M , et al. Obesity in adults: a clinical practice guideline. CMAJ. 2020;192(31):E875‐E891. 32753461 10.1503/cmaj.191707 PMC7828878

[25] Sharma AM , Kushner RF . A proposed clinical staging system for obesity. Int J Obes. 2009;33(3):289‐295. 10.1038/ijo.2009.2 19188927

[26] Padwal RS , Pajewski NM , Allison DB , Sharma AM . Using the Edmonton obesity staging system to predict mortality in a population‐representative cohort of people with overweight and obesity. CMAJ. 2011;183(14):E1059‐E1066. 21844111 10.1503/cmaj.110387 PMC3185097

[27] Müller TD , Blüher M , Tschöp MH , DiMarchi RD . Anti‐obesity drug discovery: advances and challenges. Nat Rev Drug Discov. 2022;21(3):201‐223. 34815532 10.1038/s41573-021-00337-8 PMC8609996

[28] Blüher M , Aras M , Aronne LJ , et al. New insights into the treatment of obesity. Diabetes Obes Metab. 2023;25(8):2058‐2072. 37055715 10.1111/dom.15077

[29] Wadden TA . Treatment of obesity by moderate and severe caloric restriction. Results of clinical research trials. Ann Intern Med. 1993;119(7 Pt 2):688‐693. 8363198 10.7326/0003-4819-119-7_part_2-199310011-00012

[30] Cefalu WT , Bray GA , Home PD , et al. Advances in the science, treatment, and prevention of the disease of obesity: reflections from a diabetes care editors' expert forum. Diabetes Care. 2015;38(8):1567‐1582. 26421334 10.2337/dc15-1081 PMC4831905

[31] Carlsson LMS , Sjöholm K , Jacobson P , et al. Life expectancy after bariatric surgery in the Swedish obese subjects study. N Engl J Med. 2020;383(16):1535‐1543. 33053284 10.1056/NEJMoa2002449 PMC7580786

[32] Adams TD , Davidson LE , Litwin SE , et al. Weight and metabolic outcomes 12 years after gastric bypass. N Engl J Med. 2017;377(12):1143‐1155. 28930514 10.1056/NEJMoa1700459 PMC5737957

[33] Knowler WC , Barrett‐Connor E , Fowler SE , et al. Reduction in the incidence of type 2 diabetes with lifestyle intervention or metformin. N Engl J Med. 2002;346(6):393‐403. 11832527 10.1056/NEJMoa012512 PMC1370926

[34] Tuomilehto J , Lindström J , Eriksson JG , et al. Prevention of type 2 diabetes mellitus by changes in lifestyle among subjects with impaired glucose tolerance. N Engl J Med. 2001;344(18):1343‐1350. 11333990 10.1056/NEJM200105033441801

[35] Diabetes Prevention Program Research Group . Long‐term effects of lifestyle intervention or metformin on diabetes development and microvascular complications over 15‐year follow‐up: the diabetes prevention program outcomes study. Lancet Diabetes Endocrinol. 2015;3(11):866‐875. 26377054 10.1016/S2213-8587(15)00291-0 PMC4623946

[36] Peirson L , Douketis J , Ciliska D , Fitzpatrick‐Lewis D , Ali MU , Raina P . Prevention of overweight and obesity in adult populations: a systematic review. CMAJ Open. 2014;2(4):E268‐E272. 10.9778/cmajo.20140019 PMC4251511 25485253

[37] le Roux CW , Astrup A , Fujioka K , et al. 3 years of liraglutide versus placebo for type 2 diabetes risk reduction and weight management in individuals with prediabetes: a randomised, double‐blind trial. Lancet. 2017;389(10077):1399‐1409. 28237263 10.1016/S0140-6736(17)30069-7

[38] Wilding JPH , Batterham RL , Calanna S , et al. Once‐weekly semaglutide in adults with overweight or obesity. N Engl J Med. 2021;384(11):989‐1002. 33567185 10.1056/NEJMoa2032183

[39] Qin W , Yang J , Deng C , Ruan Q , Duan K . Efficacy and safety of semaglutide 2.4 mg for weight loss in overweight or obese adults without diabetes: an updated systematic review and meta‐analysis including the 2‐year STEP 5 trial. Diabetes Obes Metab. 2024;26(3):911‐923. 38016699 10.1111/dom.15386

[40] Look AHEAD Research Group , Wing RR , Bolin P , et al. Cardiovascular effects of intensive lifestyle intervention in type 2 diabetes. N Engl J Med. 2013;369(2):145‐154. 23796131 10.1056/NEJMoa1212914 PMC3791615

[41] Lean ME , Leslie WS , Barnes AC , et al. Primary care‐led weight management for remission of type 2 diabetes (DiRECT): an open‐label, cluster‐randomised trial. Lancet. 2018;391(10120):541‐551. 29221645 10.1016/S0140-6736(17)33102-1

[42] Mingrone G , Panunzi S , De Gaetano A , et al. Bariatric surgery versus conventional medical therapy for type 2 diabetes. N Engl J Med. 2012;366(17):1577‐1585. 22449317 10.1056/NEJMoa1200111

[43] Inge TH , Courcoulas AP , Jenkins TM , et al. Five‐year outcomes of gastric bypass in adolescents as compared with adults. N Engl J Med. 2019;380(22):2136‐2145. 31116917 10.1056/NEJMoa1813909 PMC7345847

[44] Sandoval DA , Patti ME . Glucose metabolism after bariatric surgery: implications for T2DM remission and hypoglycaemia. Nat Rev Endocrinol. 2023;19(3):164‐176. 36289368 10.1038/s41574-022-00757-5 PMC10805109

[45] Panunzi S , Carlsson L , De Gaetano A , et al. Determinants of diabetes remission and glycemic control after bariatric surgery. Diabetes Care. 2016;39(1):166‐174. 26628418 10.2337/dc15-0575

[46] Blüher M . Metabolically healthy obesity. Endocr Rev. 2020;41:bnaa004. 32128581 10.1210/endrev/bnaa004 PMC7098708

[47] Klein S , Fontana L , Young VL , et al. Absence of an effect of liposuction on insulin action and risk factors for coronary heart disease. N Engl J Med. 2004;350:2549‐2557. 15201411 10.1056/NEJMoa033179

[48] Klöting N , Fasshauer M , Dietrich A , et al. Insulin‐sensitive obesity. Am J Physiol Endocrinol Metab. 2010;299:E506‐E515. 20570822 10.1152/ajpendo.00586.2009

[49] Farooqi S , O'Rahilly S . Genetics of obesity in humans. Endocr Rev. 2006;27:710‐718. 17122358 10.1210/er.2006-0040

[50] Farooqi IS , Keogh JM , Yeo GS , Lank EJ , Cheetham T , O'Rahilly S . Clinical spectrum of obesity and mutations in the melanocortin 4 receptor gene. N Engl J Med. 2003;348:1085‐1095. 12646665 10.1056/NEJMoa022050

[51] Caleyachetty R , Thomas GN , Toulis KA , et al. Metabolically healthy obese and incident cardiovascular disease events among 3.5 million men and women. J Am Coll Cardiol. 2017;70(12):1429‐1437. 28911506 10.1016/j.jacc.2017.07.763

[52] Hardy OT , Perugini RA , Nicoloro SM , et al. Body mass index‐independent inflammation in omental adipose tissue associated with insulin resistance in morbid obesity. Surg Obes Relat Dis. 2011;7:60‐67. 20678967 10.1016/j.soard.2010.05.013 PMC2980798

[53] Schmitz J , Evers N , Awazawa M , et al. Obesogenic memory can confer long‐term increases in adipose tissue but not liver inflammation and insulin resistance after weight loss. Mol Metab. 2016;5:328‐339. 27110485 10.1016/j.molmet.2015.12.001 PMC4837291

[54] Yang CH , Fagnocchi L , Apostle S , et al. Independent phenotypic plasticity axes define distinct obesity sub‐types. Nat Metab. 2022;4:1150‐1165. 36097183 10.1038/s42255-022-00629-2 PMC9499872

[55] Manolopoulos KN , Karpe F , Frayn KN . Gluteofemoral body fat as a determinant of metabolic health. Int J Obes. 2000;34:949‐959. 10.1038/ijo.2009.286 20065965

[56] Reinisch I , Ghosh A , Noé F , et al. Unveiling adipose populations linked to metabolic health in obesity. Cell Metab. 2024;S1550‐4131(24):00452‐2. 10.1016/j.cmet.2024.11.006 39694039

[57] Hinte LC , Castellano‐Castillo D , Ghosh A , et al. Adipose tissue retains an epigenetic memory of obesity after weight loss. Nature. 2024;636(8042):457‐465. 39558077 10.1038/s41586-024-08165-7 PMC11634781

[58] Lecoutre S , Rebière C , Maqdasy S , et al. Enhancing adipose tissue plasticity: progenitor cell roles in metabolic health. Nat Rev Endocrinol. 2025. doi:10.1038/s41574-024-01071 39757324

[59] Blüher M . Understanding adipose tissue dysfunction. J Obes Metab Syndr. 2024;33(4):275‐288. 39734091 10.7570/jomes24013 PMC11704217

[60] Zhang Y , Proenca R , Maffei M , Barone M , Leopold L , Friedman JM . Positional cloning of the mouse obese gene and its human homologue. Nature. 1994;372:425‐432. 7984236 10.1038/372425a0

[61] Cook KS , Min HY , Johnson D , et al. Adipsin: a circulating serine protease homolog secreted by adipose tissue and sciatic nerve. Science. 1987;237:402‐405. 3299705 10.1126/science.3299705

[62] Scherer PE , Williams S , Fogliano M , Baldini G , Lodish HF . A novel serum protein similar to C1q, produced exclusively in adipocytes. J Biol Chem. 1995;270:26746‐26749. 7592907 10.1074/jbc.270.45.26746

[63] Klein S , Gastaldelli A , Yki‐Järvinen H , Scherer PE . Why does obesity cause diabetes? Cell Metab. 2022;34:11‐20. 34986330 10.1016/j.cmet.2021.12.012 PMC8740746

[64] Blüher M . Adipose tissue dysfunction contributes to obesity related metabolic diseases. Best Pract Res Clin Endocrinol Metab. 2013;27:163‐177. 23731879 10.1016/j.beem.2013.02.005

[65] Fasshauer M , Blüher M . Adipokines in health and disease. Trends Pharmacol Sci. 2015;36:461‐470. 26022934 10.1016/j.tips.2015.04.014

[66] Gilani A , Stoll L , Homan EA , Lo JC . Adipose signals regulating distal organ health and disease. Diabetes. 2024;73:169‐177. 38241508 10.2337/dbi23-0005 PMC10796297

[67] Rome S . Muscle and adipose tissue communicate with extracellular vesicles. Int J Mol Sci. 2022;23:7052. 35806052 10.3390/ijms23137052 PMC9266961

[68] Marra F , Bertolani C . Adipokines in liver diseases. Hepatology. 2009;50:957‐969. 19585655 10.1002/hep.23046

[69] Hall JE , Mouton AJ , da Silva AA , et al. Obesity, kidney dysfunction, and inflammation: interactions in hypertension. Cardiovasc Res. 2021;117(8):1859‐1876. 33258945 10.1093/cvr/cvaa336 PMC8262632

[70] Parvanova A , Reseghetti E , Abbate M , Ruggenenti P . Mechanisms and treatment of obesity‐related hypertension‐part 1: mechanisms. Clin Kidney J. 2023;17(1):sfad282. 38186879 10.1093/ckj/sfad282 PMC10768772

[71] Pirro V , Roth KD , Lin Y , et al. Effects of Tirzepatide, a dual GIP and GLP‐1 RA, on lipid and metabolite profiles in subjects with type 2 diabetes. J Clin Endocrinol Metab. 2022;107(2):363‐378. 34608929 10.1210/clinem/dgab722

[72] Sanyal AJ . Past, present and future perspectives in nonalcoholic fatty liver disease. Nat Rev Gastroenterol Hepatol. 2019;16(6):377‐386. 31024089 10.1038/s41575-019-0144-8

[73] Neeland IJ , Lim S , Tchernof A , et al. Metabolic syndrome. Nat Rev Dis Primers. 2024;10(1):77. 39420195 10.1038/s41572-024-00563-5

[74] Ikramuddin S , Korner J , Lee WJ , et al. Lifestyle intervention and medical management with vs without Roux‐en‐Y gastric bypass and control of hemoglobin A1c, LDL cholesterol, and systolic blood pressure at 5 years in the diabetes surgery study. Jama. 2018;319(3):266‐278. 29340678 10.1001/jama.2017.20813 PMC5833547

[75] Horn DB , Almandoz JP , Look M . What is clinically relevant weight loss for your patients and how can it be achieved? A narrative review. Postgrad Med. 2022;134(4):359‐375. 35315311 10.1080/00325481.2022.2051366

[76] Blüher M . Adipokines⸺removing road blocks to obesity and diabetes therapy. Mol Metab. 2014;3:230‐240. 24749053 10.1016/j.molmet.2014.01.005 PMC3986498

[77] Ndumele CE , Matsushita K , Lazo M , et al. Obesity and subtypes of incident cardiovascular disease. J Am Heart Assoc. 2016;5(8):e003921. 27468925 10.1161/JAHA.116.003921 PMC5015307

[78] Holland WL , Miller RA , Wang ZV , et al. Receptor‐mediated activation of ceramidase activity initiates the pleiotropic actions of adiponectin. Nat Med. 2011;17:55‐63. 21186369 10.1038/nm.2277 PMC3134999

[79] Fabbrini E , Tamboli RA , Magkos F , et al. Surgical removal of omental fat does not improve insulin sensitivity and cardiovascular risk factors in obese adults. Gastroenterology. 2010;139(2):448‐455. 20457158 10.1053/j.gastro.2010.04.056 PMC2910849

[80] Global Burden of Metabolic Risk Factors for Chronic Diseases Collaboration (BMI Mediated Effects) , Lu Y , Hajifathalian K , et al. Metabolic mediators of the effects of body‐mass index, overweight, and obesity on coronary heart disease and stroke: a pooled analysis of 97 prospective cohorts with 1·8 million participants. Lancet. 2014;383:970‐983. 24269108 10.1016/S0140-6736(13)61836-X PMC3959199

[81] Calle EE , Rodriguez C , Walker‐Thurmond K , Thun MJ . Overweight, obesity, and mortality from cancer in a prospectively studied cohort of U.S. adults. N Engl J Med. 2003;348(17):1625‐1638. 12711737 10.1056/NEJMoa021423

[82] Perry RJ , Shulman GI . Mechanistic links between obesity, insulin, and cancer. Trends Cancer. 2020;6(2):75‐78. 32061306 10.1016/j.trecan.2019.12.003 PMC7214048

[83] Kahan S , Manson JE . Obesity treatment, beyond the guidelines: practical suggestions for clinical practice. JAMA. 2019;321(14):1349‐1350. 30896727 10.1001/jama.2019.2352

[84] Kaplan LM , Golden A , Jinnett K , et al. Perceptions of barriers to effective obesity care: results from the national ACTION study. Obesity (Silver Spring). 2018;26(1):61‐69. 29086529 10.1002/oby.22054

[85] Dietz WH , Baur LA , Hall K , et al. Management of obesity: improvement of health‐care training and systems for prevention and care. Lancet. 2015;385(9986):2521‐2533. 25703112 10.1016/S0140-6736(14)61748-7

[86] Butsch WS , Kushner RF , Alford S , Smolarz BG . Low priority of obesity education leads to lack of medical students' preparedness to effectively treat patients with obesity: results from the U.S. medical school obesity education curriculum benchmark study. BMC Med Educ. 2020;20(1):23. 31992274 10.1186/s12909-020-1925-z PMC6988262

[87] Rogge MM , Merrill E . Obesity education for nurse practitioners: perspectives from nurse practitioner faculty. J Am Assoc Nurse Pract. 2013;25(6):320‐328. 24170597 10.1111/j.1745-7599.2012.00785.x

[88] United States Food and Drug Administration . Guidance for industry developing products for weight management: U.S. Department of Health and Human Services, Food and Drug Administration, Center for Drug Evaluation and Research (CDER). 2007 https://www.fda.gov/media/71252/download

[89] Douketis JD , Macie C , Thabane L , et al. Systematic review of long‐term weight loss studies in obese adults: clinical significance and applicability to clinical practice. Int J Obes. 2005;29(10):1153‐1167. 10.1038/sj.ijo.0802982 15997250

[90] Unick JL , Beavers D , Jakicic JM , et al. Effectiveness of lifestyle interventions for individuals with severe obesity and type 2 diabetes: results from the Look AHEAD trial. Diabetes Care. 2011;34(10):2152‐2157. 21836103 10.2337/dc11-0874 PMC3177753

[91] American Diabetes Association Professional Practice Committee . Obesity and weight management for the prevention and treatment of type 2 diabetes: standards of care in diabetes‐2024. Diabetes Care. 2024;47(Suppl. 1):S145‐S157. 38078578 10.2337/dc24-S008 PMC10725806

[92] Lincoff AM , Brown‐Frandsen K , Colhoun HM , et al. Semaglutide and cardiovascular outcomes in obesity without diabetes. N Engl J Med. 2023;389(24):2221‐2232. 37952131 10.1056/NEJMoa2307563

[93] Sorli C , Harashima SI , Tsoukas GM , et al. Efficacy and safety of once‐weekly semaglutide monotherapy versus placebo in patients with type 2 diabetes (SUSTAIN 1): a double‐blind, randomised, placebo‐controlled, parallel‐group, multinational, multicentre phase 3a trial. Lancet Diabetes Endocrinol. 2017;5(4):251‐260. 28110911 10.1016/S2213-8587(17)30013-X

[94] Rosenstock J , Wysham C , Frías JP , et al. Efficacy and safety of a novel dual GIP and GLP‐1 receptor agonist tirzepatide in patients with type 2 diabetes (SURPASS‐1): a double‐blind, randomised, phase 3 trial. Lancet. 2021;398(10295):143‐155. 34186022 10.1016/S0140-6736(21)01324-6

[95] Wing RR , Lang W , Wadden TA , et al. Look AHEAD research group. Benefits of modest weight loss in improving cardiovascular risk factors in overweight and obese individuals with type 2 diabetes. Diabetes Care. 2011;34(7):1481‐1486. 21593294 10.2337/dc10-2415 PMC3120182

[96] Gregg EW , Jakicic JM , Blackburn G , et al. Association of the magnitude of weight loss and changes in physical fitness with long‐term cardiovascular disease outcomes in overweight or obese people with type 2 diabetes: a post‐hoc analysis of the Look AHEAD randomised clinical trial. Lancet Diabetes Endocrinol. 2016;4(11):913‐921. 27595918 10.1016/S2213-8587(16)30162-0 PMC5094846

[97] Marso SP , Daniels GH , Brown‐Frandsen K , et al. Liraglutide and cardiovascular outcomes in type 2 diabetes. N Engl J Med. 2016;375(4):311‐322. 27295427 10.1056/NEJMoa1603827 PMC4985288

[98] Gerstein HC , Colhoun HM , Dagenais GR , et al. Dulaglutide and cardiovascular outcomes in type 2 diabetes (REWIND): a double‐blind, randomised placebo‐controlled trial. Lancet. 2019;394(10193):121‐130. 31189511 10.1016/S0140-6736(19)31149-3

[99] Jastreboff AM , Aronne LJ , Ahmad NN , et al. Tirzepatide once weekly for the treatment of obesity. N Engl J Med. 2022;387(3):205‐216. 35658024 10.1056/NEJMoa2206038

[100] Sjöström CD , Lissner L , Wedel H , Sjöström L . Reduction in incidence of diabetes, hypertension and lipid disturbances after intentional weight loss induced by bariatric surgery: the SOS intervention study. Obes Res. 1999;7(5):477‐484. 10509605 10.1002/j.1550-8528.1999.tb00436.x

[101] Calle EE , Thun MJ , Petrelli JM , Rodriguez C , Heath CW Jr . Body‐mass index and mortality in a prospective cohort of U.S. adults. N Engl J Med. 1999;341(15):1097‐1105. 10511607 10.1056/NEJM199910073411501

[102] Bogers RP , Bemelmans WJ , Hoogenveen RT , et al. Association of overweight with increased risk of coronary heart disease partly independent of blood pressure and cholesterol levels: a meta‐analysis of 21 cohort studies including more than 300 000 persons. Arch Intern Med. 2007;167(16):1720‐1728. 17846390 10.1001/archinte.167.16.1720

[103] Yusuf S , Hawken S , Ounpuu S , et al. Obesity and the risk of myocardial infarction in 27,000 participants from 52 countries: a case‐control study. Lancet. 2005;366(9497):1640‐1649. 16271645 10.1016/S0140-6736(05)67663-5

[104] Shinton R , Shipley M , Rose G . Overweight and stroke in the Whitehall study. J Epidemiol Community Health. 1991;45(2):138‐142. 2072073 10.1136/jech.45.2.138 PMC1060731

[105] Emdin CA , Khera AV , Natarajan P , et al. Genetic association of waist‐to‐hip ratio with cardiometabolic traits, type 2 diabetes, and coronary heart disease. JAMA. 2017;317(6):626‐634. 28196256 10.1001/jama.2016.21042 PMC5571980

[106] Klarin D , Zhu QM , Emdin CA , et al. Genetic analysis in UK biobank links insulin resistance and transendothelial migration pathways to coronary artery disease. Nat Genet. 2017;49(9):1392‐1397. 28714974 10.1038/ng.3914 PMC5577383

[107] Koskinas KC , Van Craenenbroeck EM , Antoniades C , et al. Obesity and cardiovascular disease: an ESC clinical consensus statement. Eur Heart J. 2024;45(38):4063‐4098. 39210706 10.1093/eurheartj/ehae508

[108] Ge L , Sadeghirad B , Ball GDC , et al. Comparison of dietary macronutrient patterns of 14 popular named dietary programmes for weight and cardiovascular risk factor reduction in adults: systematic review and network meta‐analysis of randomised trials. BMJ. 2020;369:m696. 32238384 10.1136/bmj.m696 PMC7190064

[109] Carlsson LMS , Carlsson B , Jacobson P , et al. Mortality in relation to diabetes remission in Swedish obese subjects‐a prospective cohort study. Int J Surg. 2024;110(10):6581‐6590. 38896851 10.1097/JS9.0000000000001807 PMC11487030

[110] Ryan DH , Lingvay I , Deanfield J , et al. Long‐term weight loss effects of semaglutide in obesity without diabetes in the SELECT trial. Nat Med. 2024;30(7):2049‐2057. 38740993 10.1038/s41591-024-02996-7 PMC11271387

[111] Butler J , Shah SJ , Petrie MC , et al. Semaglutide versus placebo in people with obesity‐related heart failure with preserved ejection fraction: a pooled analysis of the STEP‐HFpEF and STEP‐HFpEF DM randomised trials. Lancet. 2024;403(10437):1635‐1648. 38599221 10.1016/S0140-6736(24)00469-0 PMC11317105

[112] Packer M , Zile MR , Kramer CM , et al. Tirzepatide for heart failure with preserved ejection fraction and obesity. N Engl J Med. 2024;392(5):427‐437. doi:10.1056/NEJMoa2410027 39555826

[113] Kong X , Wang M , Jiang Y . Global burden of atrial fibrillation attributable to high body mass index from 1990 to 2021: findings from the global burden of disease study 2021. BMC Cardiovasc Disord. 2024;24(1):542. 39379831 10.1186/s12872-024-04202-5 PMC11459850

[114] Donnellan E , Wazni OM , Elshazly M , et al. Impact of bariatric surgery on atrial fibrillation type. Circ Arrhythm Electrophysiol. 2020;13(2):e007626. 31940441 10.1161/CIRCEP.119.007626

[115] Reichmann R , Schulze MB , Pischon T , Weikert C , Aleksandrova K . Biomarker signatures associated with ageing free of major chronic diseases: results from a population‐based sample of the EPIC‐Potsdam cohort. Age Ageing. 2024;53(Suppl. 2):ii60‐ii69. 38745490 10.1093/ageing/afae041

[116] SantaCruz‐Calvo S , Bharath L , Pugh G , et al. Adaptive immune cells shape obesity‐associated type 2 diabetes mellitus and less prominent comorbidities. Nat Rev Endocrinol. 2022;18(1):23‐42. 34703027 10.1038/s41574-021-00575-1 PMC11005058

[117] Tabák AG , Jokela M , Akbaraly TN , Brunner EJ , Kivimäki M , Witte DR . Trajectories of glycaemia, insulin sensitivity, and insulin secretion before diagnosis of type 2 diabetes: an analysis from the Whitehall II study. Lancet. 2009;373(9682):2215‐2221. 19515410 10.1016/S0140-6736(09)60619-X PMC2726723

[118] Vrieze A , Van Nood E , Holleman F , et al. Transfer of intestinal microbiota from lean donors increases insulin sensitivity in individuals with metabolic syndrome. Gastroenterology. 2012;143(4):913‐9136. 22728514 10.1053/j.gastro.2012.06.031

[119] Aminian A , Vidal J , Salminen P , et al. Late relapse of diabetes after bariatric surgery: not rare, but not a failure. Diabetes Care. 2020;43(3):534‐540. 31974105 10.2337/dc19-1057

[120] Riddle MC , Cefalu WT , Evans PH , et al. Consensus report: definition and interpretation of remission in type 2 diabetes. Diabet Med. 2022;39(3):e14669. 34460965 10.1111/dme.14669

[121] Kunutsor SK , Zaccardi F , Balasubramanian VG , et al. Glycaemic control and macrovascular and microvascular outcomes in type 2 diabetes: systematic review and meta‐analysis of cardiovascular outcome trials of novel glucose‐lowering agents. Diabetes Obes Metab. 2024;26(5):1837‐1849. 38379094 10.1111/dom.15500

[122] Khunti K . SGLT2 inhibitors in people with and without T2DM. Nat Rev Endocrinol. 2021;17(2):75‐76. 33293703 10.1038/s41574-020-00453-2

[123] Vale C , Lourenço IM , Jordan G , et al. Early combination therapy with SGLT2i and GLP‐1 RA or dual GIP/GLP‐1 RA in type 2 diabetes. Diabetes Obes Metab. 2025;27(2):468‐481. 39604324 10.1111/dom.16077

[124] Choudhury A , Rajaram R , Sarin SK . Acute‐on‐chronic liver failure in metabolic dysfunction‐associated fatty liver disease patients: a disease multiplier. Hepatol Int. 2024;18(Suppl. 2):941‐958. 39107615 10.1007/s12072-024-10711-4

[125] Staltner R , Burger K , Baumann A , Bergheim I . Fructose: a modulator of intestinal barrier function and hepatic health? Eur J Nutr. 2023;62(8):3113‐3124. 37596353 10.1007/s00394-023-03232-7 PMC10611622

[126] Vilar‐Gomez E , Yasells‐Garcia A , Martinez‐Perez Y , et al. Development and validation of a noninvasive prediction model for nonalcoholic steatohepatitis resolution after lifestyle intervention. Hepatology. 2016;63(6):1875‐1887. 26849287 10.1002/hep.28484

[127] Yaskolka Meir A , Rinott E , Tsaban G , et al. Effect of green‐Mediterranean diet on intrahepatic fat: the DIRECT PLUS randomised controlled trial. Gut. 2021;70(11):2085‐2095. 33461965 10.1136/gutjnl-2020-323106 PMC8515100

[128] Loomba R , Abdelmalek MF , Armstrong MJ , et al. Semaglutide 2.4 mg once weekly in patients with non‐alcoholic steatohepatitis‐related cirrhosis: a randomised, placebo‐controlled phase 2 trial. Lancet Gastroenterol Hepatol. 2023;8(6):511‐522. 36934740 10.1016/S2468-1253(23)00068-7 PMC10792518

[129] Loomba R , Hartman ML , Lawitz EJ , et al. Tirzepatide for metabolic dysfunction‐associated steatohepatitis with liver fibrosis. N Engl J Med. 2024;391(4):299‐310. 38856224 10.1056/NEJMoa2401943

[130] Petta S , Targher G , Romeo S , et al. The first MASH drug therapy on the horizon: current perspectives of resmetirom. Liver Int. 2024;44(7):1526‐1536. 38578141 10.1111/liv.15930

[131] Feng G , Han Y , Yang W , et al. Recompensation in MASLD‐related cirrhosis via metabolic bariatric surgery. Trends Endocrinol Metab. 2024;S1043‐2760(24):00159‐0. 10.1016/j.tem.2024.05.009 38908982

[132] Newsome PN , Sanyal AJ , Engebretsen KA , et al. Semaglutide 2.4 mg in participants with metabolic dysfunction‐associated steatohepatitis: baseline characteristics and Design of the Phase 3 ESSENCE trial. Aliment Pharmacol Ther. 2024;60(11–12):1525‐1533. 39412509 10.1111/apt.18331 PMC11599791

[133] Lysaght J , Conroy MJ . The multifactorial effect of obesity on the effectiveness and outcomes of cancer therapies. Nat Rev Endocrinol. 2024;20:701‐714. doi:10.1038/s41574-024-01032-5 39313571

[134] Tang C , Castillon VJ , Waters M , et al. Obesity‐dependent selection of driver mutations in cancer. Nat Genet. 2024;56:2318‐2321. doi:10.1038/s41588-024-01969-3 39468367 PMC11549034

[135] Luo J , Hendryx M , Manson JE , et al. Intentional weight loss and obesity‐related cancer risk. JNCI Cancer Spectr. 2019;3(4):pkz054. 31737862 10.1093/jncics/pkz054 PMC6795232

[136] Sjöström L , Gummesson A , Sjöström CD , et al. Effects of bariatric surgery on cancer incidence in obese patients in Sweden (Swedish obese subjects study): a prospective, controlled intervention trial. Lancet Oncol. 2009;10(7):653‐662. 19556163 10.1016/S1470-2045(09)70159-7

[137] Kristensson FM , Andersson‐Assarsson JC , Peltonen M , et al. Breast cancer risk after bariatric surgery and influence of insulin levels: a nonrandomized controlled trial. JAMA Surg. 2024;159(8):856‐863. 38748431 10.1001/jamasurg.2024.1169 PMC11097101

[138] Jastreboff AM , le Roux CW , Stefanski A , et al. Tirzepatide for obesity treatment and diabetes prevention. N Engl J Med. 2024. doi:10.1056/NEJMoa2410819 39536238

